# Inferring the Effects of Protein Variants on Protein–Protein Interactions with Interpretable Transformer Representations

**DOI:** 10.34133/research.0219

**Published:** 2023-09-11

**Authors:** Zhe Liu, Wei Qian, Wenxiang Cai, Weichen Song, Weidi Wang, Dhruba Tara Maharjan, Wenhong Cheng, Jue Chen, Han Wang, Dong Xu, Guan Ning Lin

**Affiliations:** ^1^ Shanghai Mental Health Center, Shanghai Jiao Tong University School of Medicine, School of Biomedical Engineering, Shanghai Jiao Tong University, Shanghai, China.; ^2^ Shanghai Key Laboratory of Psychotic Disorders, Shanghai, China.; ^3^ School of Information Science and Technology, Institute of Computational Biology, Northeast Normal University, Changchun, China.; ^4^ Department of Electrical Engineering and Computer Science, University of Missouri, Columbia, MO 65211, USA.; ^5^ Christopher S. Bond Life Sciences Center, University of Missouri, Columbia, MO 65211, USA.

## Abstract

Identifying pathogenetic variants and inferring their impact on protein–protein interactions sheds light on their functional consequences on diseases. Limited by the availability of experimental data on the consequences of protein interaction, most existing methods focus on building models to predict changes in protein binding affinity. Here, we introduced MIPPI, an end-to-end, interpretable transformer-based deep learning model that learns features directly from sequences by leveraging the interaction data from IMEx. MIPPI was specifically trained to determine the types of variant impact (increasing, decreasing, disrupting, and no effect) on protein–protein interactions. We demonstrate the accuracy of MIPPI and provide interpretation through the analysis of learned attention weights, which exhibit correlations with the amino acids interacting with the variant. Moreover, we showed the practicality of MIPPI in prioritizing de novo mutations associated with complex neurodevelopmental disorders and the potential to determine the pathogenic and driving mutations. Finally, we experimentally validated the functional impact of several variants identified in patients with such disorders. Overall, MIPPI emerges as a versatile, robust, and interpretable model, capable of effectively predicting mutation impacts on protein–protein interactions and facilitating the discovery of clinically actionable variants.

## Introduction

Protein–protein interaction (PPI) plays a fundamental role in cellular functions and activities [1]. However, single-point missense mutations may disrupt existing PPIs or form new PPIs, which could be relevant to diseases and therapeutic strategies [2,3]. Quantifying these effects on PPIs involves assessing the changes in binding affinity caused by mutations [4]. Usually, low-throughput experiments can accurately measure these effects, but these experimental techniques are expensive and time-consuming [5]. Various computational methods, such as SIFT [6], Polyphen2 [7], CADD [8], and MutPred [9], have been developed to predict the deleteriousness of single-nucleotide variants (SNVs), especially missense variants on a protein [10]. Although these computational techniques help bridge the gap between genotype and phenotype [11], their ability is limited to predicting how a variant impacts a single gene or protein; they cannot predict how a variant can impact a protein complex or PPI. Recently, some studies focused on predicting the impact of mutations on protein interaction binding affinity [4,5,12–18], which only provide PPI binding affinity predictions as continuous values rather than as mutation effect types, such as disruption or no effects. However, there is currently no established threshold or standard to determine the protein manifestation that corresponds with ΔΔ*G*, and categorizing the effect of mutations on PPIs by category would provide a more straightforward and simplified approach to clinical assistance such as drug guidance. Furthermore, most of these techniques require protein structure information to make predictions, but only a limited number of protein complexes have experimentally validated structure data, and predicted structures may not be accurate. Some sequence-based methods do not need structural information [19,20], but their generalization ability needs further improvement because of the redundancy and small amount of training data.

**Fig. 1.**
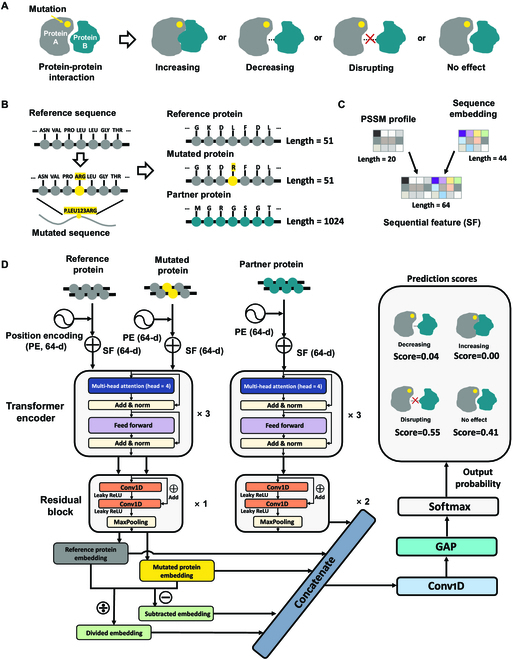
Overview of MIPPI. (A) MIPPI is designed to classify variant impacts on PPI as one of the 4 following categories: “increasing,” “decreasing,” “disrupting,” and “no effect.” (B) Unmutated (reference) as well as mutated sequences of the protein impacted by the missense mutation were cropped into a fragment with a length of 51 residues centered on the mutation site. Then, the first 1,024 residues of the partner protein were also designed as the input. If a partner protein were shorter than 1,024 residues, a series of 0s would be used as padding following the natural sequence until the length was sufficient. (C) Each position in the reference sequences, the mutated sequences, or the partner sequences was encoded as a 64D sequential feature (SF) spliced by a 20D PSSM profile and a 44D embedding vector. (D) Architecture of MIPPI. All the encoded protein sequences were fed into a deep learning framework. For a given mutation, MIPPI first provides each protein sequence position a 64D position encoding (PE) feature and concatenates it with the SF, making each input feature a 128D vector. Next, all categories of features are input into a deep neural network, which includes cascaded transformer encoders, residual blocks, a 1D convolutional layer, a GAP layer, and a SoftMax layer. Finally, MIPPI returns probabilities of all 4 output mutation impact categories to determine which one would be the most probable outcome.

To address these challenges, we leveraged the experimentally validated data provided by the IMEx consortium [21] and developed a new method, mutation impact on protein–protein interaction (MIPPI), a predictor based on a deep learning network that only requires sequence information, to make predictions. The IMEx dataset contains a set of validated experimental results on how the interaction strength changes when a mutation affects a protein in a PPI pair. Our proposed algorithm, MIPPI, is the first mutation impact prediction tool that only uses protein sequence and evolutionary conservation information to perform categorical mutation impact prediction (increasing, decreasing, disrupting, and no effect; Fig. 1A) on PPI without relying on any protein structure information. The interpretability of MIPPI also shows insights into the underlying features and mechanisms driving the predictions.

MIPPI achieved promising performance in cross-validation and outperformed the typical machine learning methods. Moreover, we delved into the analysis of selected special entries in the IMEx dataset and observed MIPPI’s capability in locating the functional mutations. To further illustrate the practicality of MIPPI, we conducted additional evaluations using missense mutation data from the neuropsychiatric mutation database of PsyMuKB [22]. The results demonstrate MIPPI’s potential in determining whether a pathogenic mutation disrupts specific PPI pairs. The co-immunoprecipitation (Co-IP) results also confirmed the effectiveness of MIPPI in predicting the effect of mutation on real-world PPIs. All the services as well as the standalone tool of MIPPI are freely accessible at the web server https://www.psymukb.net/MIPPI.

## Results

### The architecture of MIPPI

MIPPI is a supervised deep learning-based method that provides the probability of the pathogenicity of amino acid substitutions by focusing on how a missense mutation on a protein would affect the consequence of the PPI. This is achieved by predicting one of the following as the outcome of the mutation effect type on PPI: strengthens the interaction (“increasing”), reduces the interaction (“decreasing”), suspends the interaction (“disrupting”), and does not affect the interaction (“no effect”). These 4 types of mutation effects were defined by the IMEx dataset [21] (Fig. 1A, released version: 2020 May 20), which contained a set of experimentally validated variant impacts on molecular interactions (*N* = 28,000). By restricting an interacting protein pair carrying only one variation, we obtained a list of 16,451 fully annotated events about the mutation impact on specific PPI consequences.

First, we encoded the input using 3 protein sequences from PPI data: the reference (unmutated) sequence segment with the length of 51 amino acids (AA) centered around the variation position, the mutant sequence segment with the length of 51 AA centered around the missense position, and the full sequence of the PPI partner protein with the length of 1,024 AA without any mutational changes (Fig. 1B). To capture the underlying information of PPI proteins, we generated 2 types of features, i.e., “PSSM profile” and “sequence token embedding” from the primary sequences of all input proteins (Fig. 1C).

Next, a deep transformer-based residual network was constructed to associate the outcome of the impact of each variation with one of the 4 classes (Fig. 1A) in a hierarchical manner. The network was composed of 2 parallel branches, corresponding to the mutant protein and interacting partner (Fig. 1D), and then concatenated by transformer encoders, followed by residual blocks, a one-dimensional (1D) convolutional layer, a global average pooling (GAP) [23] layer, and a SoftMax [24] layer to predict the final results. For the mutant branch in the deep transformer network, we input the original as well as mutant sequences of the protein carrying the variant and passed them through the transformer encoders (Fig. 1D). For the interacting protein partner branch, only the original sequence without any variant was fed into the model pipeline.

After hyperparameter evaluations, each network branch contains a set of 3 transformer encoders, followed by a residual block in the mutated protein branch and 2 residual blocks in the interacting partner branch. Through these 2 branches, we obtained 3 output representation vectors of reference protein, mutated protein, and partner protein. Next, the 2 output vectors of the mutation branch were subtracted and divided to generate 2 auxiliary vectors to amplify the difference between the mutant branch and the reference branch after feature extraction. Then, we merged these 5 vectors and fed them into a convolutional layer and a GAP layer to reduce the risk of overfitting during the training of MIPPI. Finally, the SoftMax function output 4 scores of each class membership ranging between 0 and 1, representing the classification output’s probability distribution (Fig. 1D). Higher scores reflect a higher probability of belonging to the current classification.

### Evaluation of predictor performance

To assess the performance of MIPPI, we compared it to 5 widely used machine learning methods, including logistic regression (LR), random forest (RF), naïve Bayes (NB), K-nearest neighbors (KNN), and XGBoost. To perform an unbiased comparison, we trained all compared methods the same way as MIPPI with the most optimized hyperparameters (Table S1). We then performed a 5-fold cross-validation for the mutation effect prediction of each category (Table 1). Among the 4 categories of outcome prediction, our MIPPI model achieved the highest F1 score in 3 (disrupting: F1 = 0.657; decreasing: F1 = 0.584; and no effect: F1 = 0.813), with the “increasing” category (F1 = 0.480, slightly behind XGBoost with F1 = 0.518) being the exception. In addition, we observed that MIPPI performed among the best in overall accuracy (ACC = 0.684), followed by XGBoost (ACC = 0.668) for the 4-class classification. The t-distributed stochastic neighbor embedding (t-SNE) embedding of the model output of the GAP layer also represented good representation power of MIPPI in a 4-class prediction task (Fig. S1).

**Table 1.** MIPPI and multiple machine learning method estimation on 5-fold cross-validation with grid search.

	Class	Precision	Recall	F1 score	4-class ACC	2-class ACC	2-class MCC
Logistic regression	Disrupting	63.5	7.6	13.5	41.2	59.9	36.9
	Decreasing	51.8	9.0	15.3			
	No effect	40.5	97.6	57.3			
	Increasing	24.5	15.2	18.7			
KNN	Disrupting	84.6	11.9	20.9	46.2	73.6	55.1
	Decreasing	75.8	18.0	29.1			
	No effect	41.8	98.8	58.7			
	Increasing	56.1	29.3	38.4			
Naïve Bayes	Disrupting	56.8	16.3	25.1	41.8	53.6	18.5
	Decreasing	41.4	28.5	33.7			
	No effect	42.6	78.9	55.3			
	Increasing	13.2	12.6	12.9			
Random forest	Disrupting	**85.9**	8.8	16.0	43.7	71.1	52.5
	Decreasing	**77.9**	11.6	20.2			
	No effect	40.0	**99.5**	57.1			
	Increasing	**72.9**	26.7	38.9			
XGBoost	Disrupting	73.7	54.8	62.8	66.8	80.8	63.6
	Decreasing	67.2	50.0	57.3			
	No effect	64.0	92.5	75.7			
	Increasing	60.9	45.2	**51.8**			
MIPPI	Disrupting	65.1	**66.3**	**65.7**	**68.4**	**86.6**	**70.8**
	Decreasing	61.1	**55.9**	**58.4**			
	No effect	**81.6**	81.1	**81.3**			
	Increasing	43.5	**53.8**	48.0			

Numbers in bold indicate the best experimental results.

Next, we performed the classification on a 2-class label instead of a 4-class label. To do so, we divided the IMEx variant impact outcome data into 2 classes: an “effective class” that contained “increasing,” “decreasing,” and “disrupting” PPIs, and a “noneffective class” that contained “no effect” PPI set. As a result, we observed that MIPPI showed the best prediction outcomes with Matthew correlation coefficient (MCC) of 0.708 and ACC of 0.866, followed by XGBoost (MCC = 0.636, ACC = 0.808; Table 1 and Table S2). In summary, MIPPI achieved the best results of most metrics in multiclass as well as binary classifications, demonstrating that it would be an effective and robust method.

**Fig. 2.**
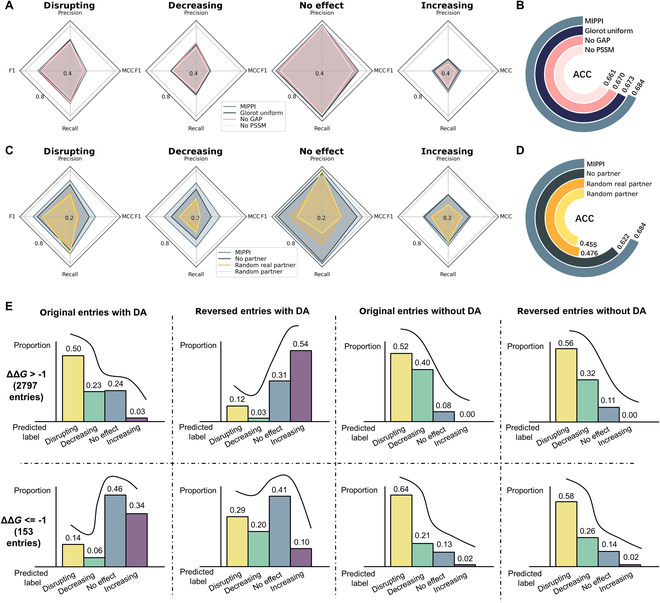
Ablation study and the evaluation of the data augmentation strategy. (A) Ablation study to test the model contribution of different components of MIPPI. The He uniform initialization layer [25] was replaced by the Glorot uniform initialization layer [26], the GAP layer was replaced by a fully connected layer, and the input feature of PSSM was removed from the model separately. Detailed substructure test performance is presented in Table S5. (B) The ablation study showed the accuracy contribution of the He uniform initialization layer, the GAP layer, and the input feature of PSSM. (C) The ablation study of the partner protein verified the necessity of inputting the real partner protein to MIPPI. The “MIPPI-no partner” model was trained when the input of the PPI partner was removed. The “MIPPI-random real partner” model was trained by randomly selecting a protein in the original training set as the PPI partner input of MIPPI. The “MIPPI-random partner” model was trained by taking randomized sequences (not real protein sequences) as the PPI partner input of MIPPI. (D) In the same 2-class classification as (B), the ablation study showed the accuracy contribution of various input strategies for PPI partner information. (E) Proportions of ΔΔ*G* entries predicted by MIPPI on the original and reversed entries of the SKEMPI v2 dataset described in Fig. S6, with data augmentation (DA) and without data augmentation (no DA). The upper panel shows the prediction results of the entries with ΔΔ*G* > −1 kcal/mol (2,797 entries), and the lower panel shows the ones with ΔΔ*G* ≤ −1 kcal/mol.

During the model construction, we have conducted several model tuning strategies to improve the performance, including adding the He initialization layer [25], GAP layer, Position-Specific Scoring Matrix (PSSM) profiles as the input feature, and PPI partner sequences as the model input. Here, we performed an ablation study to evaluate the contribution of each applied tuning strategy to the final prediction in the 4-class as well as 2-class classification tasks on 4-fold cross-validation (Fig. 2A to D). We observed that the performance using the Glorot uniform initialization layer [26] decreased in all category tests compared to the He uniform initialization layer (Table S3 and Fig. 2A and B). When replacing the GAP layer with a fully connected layer at the end of the model, or removing the PSSM profiles from the input of MIPPI, the performance also decreased.

Even for the same missense mutation, the final effect on the PPI may be different for different interaction partners. Therefore, we included the information on the partner protein when we constructed the MIPPI training model. Here, we performed 3 ablation tests to evaluate the contribution of an individual component to the prediction (Table S3 and Fig. 2C and D). First, we trained the “MIPPI-no partner” model by removing the PPI partner input branch in the model structure using the same training protocol of MIPPI. As expected, the modified model did not perform as well as the original MIPPI model in all categories (Fig. 2C), and the ACC of the 2-class classification task decreased from 0.684 to 0.622 (Fig. 2D). Second, we generated mock sequences using random letters as the PPI partner input of MIPPI and evaluated the model using a 5-fold cross-validation. The results showed that the model’s prediction performance with mock partner sequences decreased drastically compared with the original MIPPI model (Fig. 2C and D and Table S3). In addition, we randomly selected proteins from MIPPI’s original training set as the partner protein and retrained the model. We observed that the prediction performance of the model was slightly better than the model with mock partner sequences but still far worse than the original MIPPI model.

To validate the effectiveness of transformer blocks in the model, we also conducted an ablation experiment by keeping the other components unchanged while replacing the transformer layers with fully connected layers, 1D convolutional layers, or long short-term memory (LSTM) layers, respectively (Fig. S9 and Table S3). We observed that the transformer blocks used in MIPPI outperformed all other compared components, indicating its effectiveness in capturing both local and long-range feature relationships.

### The modified data augmentation strategy increased the model’s robustness

It was reported that owing to the imbalanced and biased training data and the overfitting during training, many machine learning-based algorithms would give random and erroneous prediction results when exchanging the mutant branch and the reference branch of input [27]. To correct for potential biases introduced by the data imbalance, we first considered 2 commonly used strategies for imbalanced data learning: Tomek Links [28] for undersampling and SMOTE [29] for oversampling. However, we found that Tomek Link was not applicable to our data pattern because of the limited sample sizes, especially in the category “increasing,” and SMOTE would produce additional pseudo samples without clear, meaningful labels.

It has been shown that using the hypothetical reverse mutations strategy [5] is an excellent way to balance and increase the sampling of the dataset without adding new proteins. It could provide an additional validation step to detect possible overfitting in the prediction models [30,31]. Thus, we applied hypothetical reverse mutation, which assumes that the change of PPI binding affinity when a mutation happens on a wild-type protein would be equivalent to the negative change of hypothetical reverse mutations from the mutant to its wild-type form.

Therefore, we implemented a modified data augmentation strategy by adding 2 new types of entry (reversed entry and nonmutated entry) into the initial training set for MIPPI during the model training (Fig. S6). MIPPI was trained on the DA-enhanced training set, and we evaluated the DA strategy using 5-fold cross-validation on the 4-class and 2-class classification tasks on the 5 test sets (Table S10). We observed that the MIPPI model achieved an observable performance increase on the reversed entries of the dataset compared to “MIPPI-no DA” on the 2-class classification task (ACC_MIPPI_ = 0.77 versus ACC_MIPPI-no DA_ = 0.44). In addition, MCC and F1 score of all categories of the MIPPI model also outperformed the “MIPPI-no DA” on the 4-class classification task.

To further verify the validity of the implemented data augmentation strategy, we reversed all the entries in the SKEMPI v2 dataset with the ΔΔ*G* labels remaining as an external validation set. In theory, MIPPI must give opposite prediction results on the reversed entries. So, we compared the proportion of the prediction results of MIPPI on the SKEMPI v2 dataset (2,797 entries with ΔΔ*G* > −1 kcal/mol and 153 entries with ΔΔ*G* ≤ −1 kcal/mol) before and after applying the data augmentation with original and reversed PPI input (Fig. 2E). We observed that without data augmentation, MIPPI almost maintained the original prediction results after swapping the mutant and reference sequences [Fig. 2E (5 to 8)], while after data augmentation, MIPPI predicted almost the opposite outcome compared with the results predicted before data augmentation [Fig. 2E (1 to 4)]. All these results showed our data augmentation strategy’s necessity and effectiveness.

### The interpretability of the MIPPI highlighted the critical region of the protein interaction surface

MIPPI only uses 2 independent sequences as model inputs, without information such as protein folding or docking as additional features in advance to help improve prediction accuracy while initializing the model. However, other deep learning models using sequences have shown that the attention weights would often vary along with sequences and could be used to assess the contribution of each protein residue to the outcome change prediction of PPI [32]. Therefore, to investigate how MIPPI achieved predictions with high accuracy using only protein sequences, we performed a weight-based analysis to analyze the model interpretability by tracking the distribution of attention weights.

**Fig. 3.**
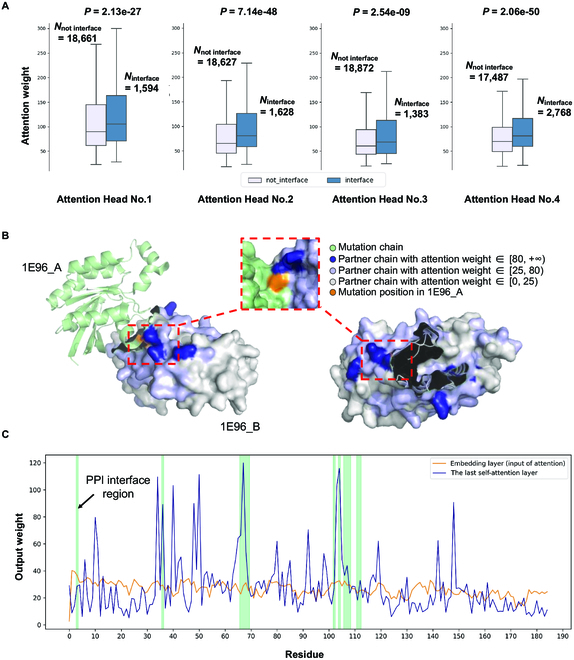
Mapping attention weights to protein sequences. (A) For each PPI partner protein in the training dataset, the top 5 residues with the highest attention weights in each attention head (Attention Head No.1 to No.4, 20,615 residues in total) were considered to explore the relationship between the attention weight of residues and their locations (in the PPI interface or not). *N*_interface_ represents the number of residues in each attention head in the PPI interface, while *N*_not interface_ represents the number of residues not located in the PPI interface. The attention weights here were exported from the last multihead self-attention layer, and the weights of padding positions were discarded. The Mann–Whitney *U* test was performed to test the distribution difference of the attention weights of residues. (B) Human signaling heterodimer (PDB structure ID: 1E96) with 2 chains (protein RAC1 as chain IE96_A and protein NCF2 as chain IE96_B) used to illustrate the weights of the second attention head of the last multihead self-attention layer from MIPPI models. RAC1 is shown in light green with a missense mutation (p.Asn26His, shown as orange). NCF2 is shown in light gray with multiple attention weights mapped on its surface (the darker the color, the higher the attention weight). (C) MIPPI model output of the attention weight distribution in 1E96_B at the residue level (total chain length: 185 AA). The green-shaded region represents the PPI interface.

It has been shown that changes to PPI interfaces can affect the formation of multiprotein complexes, leading to disruptions in PPI networks within and between cells, further causing phenotypic changes as functional interactions are created or disrupted [33,34]. Thus, to investigate whether MIPPI captured the underlying information, such as PPI interfaces, from sequences and embedded them for prediction, we first mapped the top 5 attention weights of each PPI partner generated from the neural network to the known PPI interfaces, resulting in 20,615 residues. We then performed the Mann–Whitney *U* test to compare the attention weight difference between interface and noninterface regions for each attention head. As expected, the results showed that the interface regions had significantly higher attention weights than noninterface regions in all features learner (attention head): *P* = 2.13 × 10^−27^ in attention head No.1, *P* = 7.14 × 10^−48^ in attention head No.2, *P* =2.54 × 10^−9^ in attention head No.3, and *P* =2.06 × 10^−50^ in attention head No.4 (Fig. 3A). These results indicated that the MIPPI model captured vital information for our classification task, such as the protein interaction interfaces, and tended to assign higher weights to these regions for better prediction performance, despite using only sequences.

Next, we used the interaction between protein RAC1 (Ras-related C3 botulinum toxin substrate 1) and protein NCF2 (also known as P67PHOX, neutrophil cytosol factor 2), with a missense mutation (p.Asn26His) on RAC1, as an example to evaluate if high attention weights would indeed imply meaningful functional regions from the sequences impacted by the mutation. By visualizing the missense mutation position on the chain of the mutant protein and the attention weight distribution on the chain of the interacting partner, we observed that most of the highest weights (higher than 80) appear on and around the interacting interface area (Fig. 3B and Fig. S8). Furthermore, the attention weights tended to decrease as the distance from the interface region increased. Interestingly, a region with relatively high weights (higher than 25) in the partner chain was observed close to the missense position, although all sequences were input into the model independently during model inputs.

To better illustrate the learning effect of MIPPI, we visualized the weight distribution of the partner protein before and after transformer blocks at the residue level (Fig. 3C). The architecture of MIPPI included 3 transformer blocks, with the input layer anterior and the residual blocks posterior (Fig. 1D). Here, we extracted the weights of the input layer and the multihead self-attention layer with head No.2 from the third transformer block for comparison. In contrast to the input layer, the weight curve of the multihead self-attention layer fluctuated significantly, with most peaks of weights overlapping with or near the interface region, directly reflecting MIPPI’s powerful learning ability. In sum, this case showed that the weights of the multihead self-attention layers might indicate crucial biological information, although the model input did not contain any information about protein folding and docking.

### Estimating pathogenic effects on IMEx special entries

During the model training phase, to avoid bias in model training, we filtered the initial IMEx dataset by removing the following 2 types of data: (a) entries with the mutation effects of “causing” and (b) entries with one-mutation-multiple-effects annotations. Here, to further examine the robustness and applicability of the trained MIPPI model, we reintroduced these excluded data as test inputs to estimate the pathogenic effects on these entries.

The “causing” category was defined by IMEx as a set of interactions between 2 proteins caused by missense mutations and has a sample size of 54. Given its limited size for the model training for effective classification, we excluded them from the initial training set during preprocessing (Table S6). Thus, as MIPPI has never been trained with “causing” data, the “causing” effect set would be a fitting independent test set for the robustness and biological rationality of the MIPPI model.

**Fig. 4.**
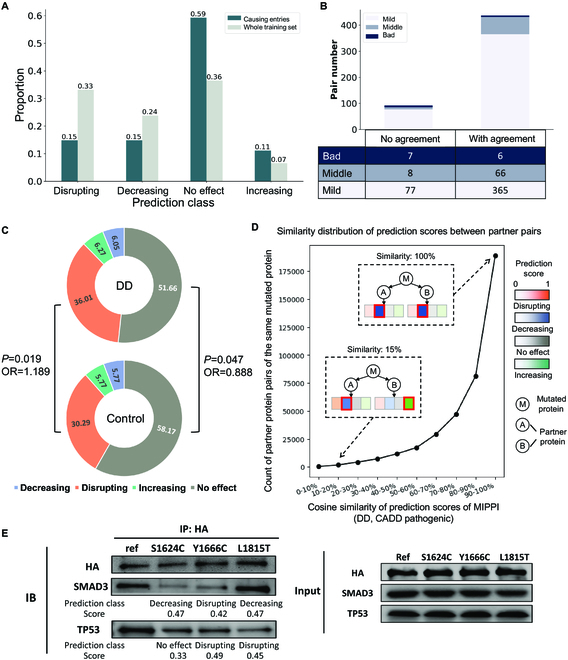
Prediction of the special entries on the IMEx dataset and model evaluation against SIFT using missense variants from PsyMuKB [22] databases. (A) Prediction results of “causing” entries, compared with the whole training dataset prediction distribution. (B) Conflicting entry prediction distribution, classified by whether they have been reported in the literature. “With agreement” means that MIPPI’s prediction result is the same as at least one of those reported references. (C) Donut charts showing the distribution of the prediction results of MIPPI on the developmental delay (DD) correlated entries and the control entries from PsyMuKB. (D) Cosine similarity distribution of the 4 prediction scores from MIPPI varied from 0 to 100% between various partner protein pairs of the same DD-correlated mutated protein from the PsyMuKB dataset; all the mutations were pathogenic with a CADD score larger than 30. There are 396,604 partner pairs from 3,051 mutated proteins with 13,237 partners. (E) Co-IP experiment results. SETD2 reference and 3 selected variants (S1624C, Y1666C, and L1815T) interact with SMAD3 and TP53, with MIPPI prediction class and score.

We observed that MIPPI showed a more neutral effect prediction on the “causing” entries test set than the prediction outcomes on the entire training set (Fig. 4A, χ^2^ *P* value = 2.14 × 10^−3^). In particular, MIPPI prediction on the “causing” test set showed 59% of impact outcomes as “no effect” and 11% of outcomes as “increasing,” which were significantly higher than prediction outcomes on the entire training set showing only 36% of impacted outcomes as “no effect” (odds ratio = 1.42, Fisher test *P* value = 1.3 × 10^−1^) and 7% as “increase” (odds ratio = 2.26, Fisher test *P* value = 5.5 × 10^−2^). Although the “causing” entries were not included in the training process of MIPPI, the model tended to consider the mutation of the “causing” entries as a positive impact on PPI, a kind of support that the model learned the pattern of the changes caused by mutation.

Given that the IMEx dataset [21] was manually curated from thousands of experimental studies under various conditions, there might be different mutation effects reported in IMEx for the same PPI carrying the same missense mutation. Thus, we excluded this type of entry with multiple mutation effect annotations from the preprocessing step before constructing the MIPPI model (see Table S6). However, to further test the robustness of MIPPI’s performance, we apply MIPPI on these PPIs with given missense mutations annotated by IMEx to estimate the impact outcome. We observed that over 80% of the predicted outcomes were shown to be one of the reported effects (Fig. 4B), suggesting that MIPPI could be used as an auxiliary judgment tool when references have conflicting statements. Another particular entry type was proteins with multiple missense mutations that caused changes to a single PPI pair. Although it is difficult to determine the contribution of each mutation on the overall impact, MIPPI prediction of each mutation separately could help detect and evaluate the functional mutations.

### Application of MIPPI’s partner-specific mutation effect prediction in developmental delay captures enriched molecular mechanisms

Pathogenicity of missenses is often measured on whether they lead to loss of stability, aberrant folding, and increased degradation of the impacted protein. However, mutations could also disrupt the functional properties of a protein complex, such as macromolecular binding (e.g., PPIs) and metal binding. Thus, one crucial advantage of MIPPI over classic prediction tools on the missense effect, such as CADD [35], SIFT [36], and PolyPhen-2 [37], is that MIPPI considers the interacting protein pair as a whole and predicts the possible impact of the mutation on the interaction according to the corresponding interacting protein, rather than only predicting the pathogenicity of the mutated protein.

Neurodevelopmental disorders, such as developmental delay (DD), have a vital genetic component [38]. Recent whole-genome and whole-exome sequencing in DD has identified thousands of de novo mutations in affected patients [39,40]. However, identifying de novo mutations that could affect the functional properties of a protein complex, such as PPIs, remains challenging. We applied MIPPI to a set of 4,228 de novo missense mutations (3,188 from DD cases, 1,040 from healthy control) from the PsyMuKB database [22] and mapped them against a set of 52,655 physical PPI partners on the BioGrid database [41] (released version: 2022 March 25) to predict the category of mutation effect. First, we observed that missenses from DD cases had a significantly larger proportion of disrupting effect on PPIs predicted than those from controls (36% versus 30% with *P*_FisherExact_ = 0.019, see Fig. 4C and Table S11). For the predicted “no effect” entries, the missenses from DD cases showed a significantly lower proportion than those from controls (52% versus 58% with *P*_FisherExact_ = 0.047; see Fig. 4C and Table S11).

To investigate the extent to which a pair of same-mutant-different-partners would have dissimilar mutation effect outcome profiles, we calculated the dissimilarity of their mutation effect profiles (cosine similarity) by comparing all 4 effect categories’ (disrupting, decreasing, increasing, no effect) prediction scores between 2 sets of PPIs with the same mutant protein. We focused on a set of 612 DD pathogenic missenses [the PHRED-scaled score of combined annotation dependent depletion (CADD) [35] ≥ 30; we predicted damaging by SIFT [36] and PolyPhen-2 [37] in the HumDiv training set] and 612 impacted proteins with all their 12,972 interacting partners.

Next, we calculated the cosine similarity (ranging from 0 to 100%) of the prediction score profiles of the mutation effect (a 4D vector output by MIPPI) by comparing all possible pairs of same-mutant-different-partner PPIs (Fig. 4D). In total, 48.45% of same-mutant-different-partner pairs exhibit nearly identical effect prediction profiles, i.e., a cosine similarity larger than 90%. Next, we found that 0.11% (427) of pairs exhibit almost distinct mutation effect prediction profiles, yielding the maximal cosine similarity of 10%. For example, gene *HDAC3* carrying a missense (g.G277A) interacts with 2 different partners, *NCOR1* and *ZBTB33*, where the predicted mutation effect profile of *HDAC3*–*NCOR1* is “increasing” and the predicted mutation effect profile of *HDAC3*–*ZBTB33* is “disrupting.” We observed that 6.58% of same-mutant-different-partner pairs have a cosine similarity < 50%, which indicates that the same deleterious mutation might have different effects on the interaction of varying PPI partners and MIPPI has the potential to explain whether a pathogenic mutation effect on a protein could lead to the disruption of a specific PPI pair.

In addition, for those genes carrying DD missenses, we investigated whether their PPI partners with different MIPPI-predicted effects would have distinct biological functions. As shown in Fig. S7 and Table S12, we found that protein partners of PPIs with MIPPI-predicted effects as disruption were enriched in pathways such as head development (adjusted log*P* = −20.73) and actin filament process (adjusted log*P* = −19.4). These results supported the value of MIPPI’s unique capacity to predict mutation effects on different PPI partners in clinical and biological studies.

### Experimental validation of the impact of missense mutations

High pathogenicity scores from MIPPI for a given mutation provide hypotheses of disruption of PPI that could lead to disease. We used the Co-IP system to test the impact of high-scoring mutations on PPIs with corresponding interacting partners.

We selected the gene *SETD2*, which has been reported to be related to neuropsychiatric diseases, such as autism [42] and Sotos-like syndrome [43], and its 2 representative de novo missense mutations, i.e., Y1666C and L1815T as well as a somatic missense mutation, i.e., S1624C reported from autism patients [42,44,45] for experimental validation. Among them, S1624C and Y1666C were also found in cancer patients [45], suggesting the importance of these 2 mutations in autism spectrum disorders (ASD). We also selected 2 other genes, *SMAD3* and *TP53*, as they were the confirmed protein-interacting partners of SETD2 [46,47]. Next, we generated 3 mutant clones of SETD2 carrying 3 mutations separately, as well as wild-type clones of SETD2. Then, we tested 2 PPIs, SETD2–SMAD3 and SETD2–TP53, for wild-type as well as mutant variants using Co-IP to compare the results (Fig. 4E).

We observed that 2 mutations, S1624C and Y1666C in the *SETD2* gene, disrupted the interaction between protein SETD2 and the partner SMAD3, consistent with MIPPI predictions that assigned “decreasing” for S1624C (score 0.47) and “disrupting” for Y1666C (score = 0.42) (Fig. 4E). SMAD3 is an intracellular molecule involved in the transforming growth factor (TGF)-β signaling cascade. It has been shown that TGF-β/SMAD3 signaling is associated with several pathological features of neurological diseases, such as dopaminergic neurodegeneration, reduction of dopaminergic axons and dendrites, α-synuclein aggregation, and modulating γ-aminobutyric acid (GABA) neurotransmission [48–51]. Furthermore, deficiencies in TGF-β/SMAD3 signaling may represent a risk factor for the development of some brain disorders [49,51,52]. The development of α-synuclein aggregates and display of dopaminergic and hippocampal dysfunction in SMAD3-deficient mice [52] suggest the importance of maintaining this PPI (SETD2–SMAD3). The experimental results for S1624C (score 0.47) and Y1666C (score 0.42) were in agreement with the predictions, although the MIPPI score for L1815T (score 0.47) did not match the observed decrease of binding. L1815T may therefore be an undiscovered pathogenic variant missed by MIPPI; alternatively, it is a PPI-altering variant that does not lead to a disease phenotype.

We then examined the effect of 3 mutations on the interaction between SETD2 and TP53. We observed that all 3 variants showed a decrease in interaction strength from the original PPI. Two mutations, Y1666C and L1815T in the SETD2 gene, disrupted the interaction between protein SETD2 and the partner protein TP53, consistent with MIPPI predictions that assigned “disrupting” for Y1666C (score 0.49) and L1815T (score 0.45). MIPPI predicted “no effect” for S1624C with a low confidence score (score 0.33), which matched the relatively weak damage to PPI caused by S1624C in the experiment (Fig. 4E). It is known that SETD2 can interact with TP53 and selectively regulate the transcription factor activity of TP53, a tumor suppressor gene [53], and the loss of SETD2 could inactivate the TP53-mediated checkpoint in cancer [54]. Similarly, the binding of SETD2 to TP53 can modulate the expression of a specific set of TP53 downstream target genes, including the apoptosis-related genes puma, noxa, and p53AIP1, which are relevant to cancer development [55]. Overall, MIPPI predictions agreed well with experimental observations.

## Discussion

It remains a fundamental challenge to accurately predict the effects of missense mutations on PPI based solely on primary sequences. To tackle this challenge, we introduced several innovations in MIPPI, enabling a comprehensive exploration of abstract features derived from protein sequences and interpreting them from the perspective of the PPI interface. Unlike previous tools that focused on PPI binding affinity changes caused by mutation, MIPPI provided a more straightforward prediction of how one mutation affects PPI with a specific partner, which offered a simplified approach to clinical assistance such as drug guidance. Benefiting from the learning ability of the deep learning network and the data augmentation strategy, MIPPI achieved the best cross-validation performance compared with multiple existing machine learning methods. Moreover, the model demonstrates remarkable generalization robustness, which is an advantage in real-world applications. In addition, with the help of the self-attention mechanism implemented in MIPPI, the deep learning network is no more a black box in capturing features but demonstrates a significant enrichment trend of higher attention weights to the regions of PPI interfaces. The interpretability of the model indicates that the important feature identified by MIPPI is precisely the information from PPI interfaces that is important for the stability of PPI, which showcases the learning ability of MIPPI, although the model input did not contain any information other than protein sequence.

Although MIPPI was trained on a filtered dataset that only contained single-mutation entries labeled “disrupting,” “decreasing,” “increasing,” and “no effect,” when dealing with the “causing” entries in the IMEx dataset, the model tended to consider these entries as a positive impact on PPI, which proves the robustness and biological rationality of the MIPPI model. In addition, we also observe that MIPPI could be used as an assessment tool when the same mutation is reported with conflict effects on the same PPI by multiple studies. Furthermore, when multiple missense variants of a protein caused changes to the PPI, though it is difficult to identify the contribution of each mutation on the overall impact, the prediction result of MIPPI for each mutation could be taken as a reference to evaluate which mutations are functional.

Some methods have been developed to predict the probability that a substitution is damaging, such as CADD [35], SIFT [36], and PolyPhen-2 [37], but these tools do not give further explanations for why a mutation is determined to be deleterious. Furthermore, for each mutated protein, various PPI partners might exist, but these deleteriousness prediction tools cannot distinguish between the distinct effects of the mutation on various PPI partners. In contrast, MIPPI might be able to explain whether a pathogenic mutation effect on a protein could lead to the disruption of a particular PPI pair. The study on in-dataset special entries indicated the ability of MIPPI to locate the functional mutations on certain occasions, and the (Co-IP) results confirmed the effectiveness of MIPPI prediction. In addition, we also provide an interactive web server for users to access our tool.

Predicting the impacts of genetic mutation on PPI at the genome-wide scale can profoundly improve the knowledge of systems biology and disease mechanisms. Biological systems work integratively and synchronously, where thousands of proteins cooperate with each other via physical interaction. Consequently, disruption of a single protein could spread across the entire protein network, leading to phenotypic impacts such as complex diseases. In line with this notion, the omnigenic model [56] supposes that for each complex phenotype, only a small number of core genes could directly control it, while a large number of mutations on other peripheral genes impact the phenotype via a gene network that indirectly affects the core genes. MIPPI provides a direct means to evaluate the omnigenic model: For mutations on genes not directly relevant to the disease, we could find its PPI partners that truly lead to the disease. By applying MIPPI at the genome-wide scale, the entire picture of the disease gene network could be systematically depicted, where we could highlight the core genes underlying the disease mechanism.

However, MIPPI has certain limitations. As predicting PPI change upon mutation on sequence-based features is very challenging and strongly depends on the dataset size and quality [57], the prediction performance still has considerable room for improvement. With the development of the IMEx dataset and other datasets with similar annotations, we believe that MIPPI will achieve better performance and generalization ability. Recently, Google released AlphaFold2 [58] and AlphaFold-Multimer [59], which could predict the structure of proteins and protein complexes using only protein sequences. As the prediction performance of AlphaFold2 and AlphaFold-Multimer in predicting the structures that carry mutations has not been comprehensively evaluated, we did not implement them as a 3D-feature generator. However, it is conceivable that incorporating structure-disrupting mutations experimental data will enable this feature in future versions of protein structure prediction programs [60], and we believe that this feature will enhance the prediction ability of MIPPI.

With the rapid evolution of deep learning, we anticipate that future methods will offer even more reliable predictions for the effects of mutations on PPI. In line with this trajectory, we are committed to advancing MIPPI by exploring and integrating more powerful deep learning architectures. Additionally, we will assess the potential benefits of incorporating predicted 3D features to further enhance prediction accuracy. Moreover, we plan to develop a more user-friendly software system from MIPPI, along with optional prediction methods in support of a variety of data formats, mutation analysis tools, and interactive visualizations. By embracing these future developments and enhancements, we aspire to make MIPPI an even more robust and valuable tool for the scientific community, enabling precise predictions and empowering researchers to investigate mutation effects on PPIs.

## Materials and Methods

### Data preprocessing

Here, we used a protein mutation dataset from the IMEx Consortium [21]. This dataset features more than 50,000 annotations describing the effect of amino acid sequence changes on physical protein interactions with tested experimental evidences. Annotations from over 297 diverse species are included, with over 60% of the events annotated in human proteins. The dataset is updated in the IntAct database (https://www.ebi.ac.uk/intact/), in which the version of 2020 May 20, was used in the research.

This dataset is human-curated from thousands of published references with duplicates and other biases. We conducted a series of preprocessing steps to filter data, including removing duplicates and dropping conflicts (Table S6). We dropped electronically annotated data because there is no exact experimental evidence for these entries. To ensure the accuracy of data labels, we selected only binary PPI items. We dropped entries with multipoint mutant protein for our research considering the single-point mutation effect on PPI. As the data were derived from various references, it brought about the problems of duplication and contradictory labeling. Duplicated entries are reserved only once, and all contradictory entries are deleted. As there are few “causing” class entries (less than 100) and mutated proteins that cause new PPI are rare in nature, putting these data into training will cause serious false-positive problems, so we deleted the data in this category.

After preprocessing, there were 16,451 entries remained. Considering the redundancy issues in this dataset, we ensured that our model input has information from 3 aspects: original protein, mutant protein, and its partner. Therefore, traditional de-redundancy methods are not applicable. To check the redundancy of our dataset, we counted the repetitions of each unique protein pair (Fig. S4A). Each repetition implies the same participants in a PPI with a different mutant. Most protein pairs were only mentioned once. Less than 7.1% of the protein pairs were repeated more than 10 times, confirming that our dataset has low redundancy.

### Feature engineering

This research tested various universal features used in similar machine learning tasks (Fig. 1C) [61]. We designed a triplet feature matrix, representing features of the original protein, mutant protein, and its partner protein. Each part corresponds to a protein’s primary amino acid sequence, consisting of 2 sets of features, protein sequence encoding and position-specific scoring matrix (PSSM). For neural networks to take fixed-size input and the task focusing on the mutated position in the affected protein, we experimentally designed a sequence length of 51 residues with the mutation in the middle for the original and mutated proteins to balance prediction accuracy with computational cost. For partner proteins, the binding sites are unknown in advance, so full-length sequences are needed. We designed the first 1,024 residues of the partner protein as input. This length covered more than 90% of partner proteins in the dataset (Fig. S4B). This length interception strategy is used for all features.

#### Amino acid sequence matrices

There is basic and nonbias information in the primary amino acid sequence to describe a protein. As mentioned above, the 51-length fixed window was used for the affected protein. For involved protein sequences with fewer than 25 residues at both sides of the mutated position, 0 was used as padding at the end of the sequences. For partner proteins shorter than 1,024 AA, 0 was used as padding after the sequence. Each amino acid in the protein sequence was represented as a token for model input. Each sequence token ranges from 0 to 20 to represent zero padding or one of the 20 standard amino acids.

#### PSSM feature

Sufficient protein conservation information has been widely used in protein structure prediction, which is highly related to PPI and other biological processes. Here, we used PSI-BLAST [62] tool to generate PSSM. In the alignment, the inclusion threshold *e* value for pairwise alignment was set to 1 × 10^−3^, using Uniref90 [63] as a search database with 3 PSI-BLAST iterations. Then, the PSSM was normalized by the sigmoid function to restrict into the range (0, 1). Each amino acid in the sequence was represented as a vector of 20 real numbers to form a 51 × 20 matrix for each affected protein and a 1,024 × 20 matrix for the partner protein. The same zero-padding strategy as amino acid sequence matrices was used, but a 20D vector for each padding position.

After the feature engineering described above, we obtained 2 types of features for all 3 parts of the model input. For the original protein and corresponding mutant protein, a 51 × 1 sequence token vector and 51 × 20 PSSM matrix were generated, while a 1,024 × 1 sequence token vector and 1,024 × 20 PSSM matrix were generated for the partner protein. Each position in the reference sequences, the mutated sequences, and the partner sequences was encoded as a 64D sequential feature (SF) spliced by a 20D PSSM profile and a 44D embedding vector. The 44D embedding vector was obtained by utilizing the “Embedding Layer” of TensorFlow based on the OneHot encoding of input sequences. All these features formed the whole multifeature input for our model.

### Model architectures

We designed a deep neural network architecture (MIPPI) for mutation impact classification, using the basic architecture of a modified transformer encoder and residual block [64] (Fig. 1D). Transformer and transformer-based network BERT has been used in various natural language processing (NLP) tasks and achieved state-of-the-art performance. The protein sequence is just like a kind of biological language. Permutations and combinations of amino acids of various lengths constitute proteins, so the transformer model is an ideal architecture for this task. Transformer can capture long-term dependencies and has better parallelism than the recurrent neural network (RNN) [65] and LSTM [66] network.

We changed the ordinary transformer sequence embedding part to adapt to our multifeature input rather than only sentence tokens in NLP tasks (Fig. 1D and Fig. S5). Protein sequence tokens were embedded into 44 dimensions and concatenated with a 20D normalized PSSM matrix to form the whole sequence embedding part. Sine and cosine functions were used in the positional embedding to capture distance dependency in sequences:

$$\mathrm{PE}\left(\mathrm{pos},2i\right)=\text{sin}\left(\frac{\mathrm{pos}}{10000^{\frac{2i}{d_{\text{model}}}}}\right)$$ (1)

$$\mathrm{PE}\left(\mathrm{pos},2i+1\right)=\text{cos}\left(\frac{\mathrm{pos}}{10000^{\frac{2i}{d_{\text{model}}}}}\right)$$ (2)

where *pos* is the position of an amino acid in the input sequence, *i* is the embedding index, and *d*_model_ is the dimension of the output embedding space.

To avoid the adverse effect of the zero-padding part in the sequence on the subsequent structure of the model, we applied zero masking to minimize the weights of the zero-padding sequence when processing the data.

The transformer encoder block mainly consists of 2 substructures: multihead attention and a feedforward network. Multihead attention in a transformer is a type of self-attention. Query (Q), key (K), and value (V) are each calculated from the input embedding, and the result from various heads will combine to give the final output. The feedforward network in MIPPI is a double-dense layer with layer normalization and dropout operation.

Each transformer block comprised a 4-head self-attention layer and a feedforward subnetwork, which is a classical structure of the transformer. The self-attention layer checks attention with all residues in the same sequence at once, which gives the interpretability to MIPPI accompanied by visualization. Concurrently, multiple heads would look into various regions simultaneously and independently, thereby enabling a comprehensive assessment of the contributions of each residue of proteins. The feedforward subnetwork in MIPPI contains 2 fully connected layers with layer normalization and dropout operation, designed to generate an element-wise nonlinear transformation of incoming vectors. Each residual block contains 2 two-layer convolutional neural network (CNN) subblocks, with a double-layer skip that has nonlinearities (ReLU), and a max pooling layer at the end of the skip in each subblock to make the effective network deeper while reducing the impact of vanishing gradients.

### Implementation details

When comparing the performance of MIPPI with other machine learning methods, validating the effectiveness of model components through ablation experiments, and assessing the efficacy of data augmentation, we used a 5-fold cross-validation to demonstrate the stability and accuracy of the methodology. After finalizing the architecture of MIPPI and establishing the data augmentation strategy, considering the simplicity in subsequent analysis and visualization, we divided the whole dataset into training, validation, and test sets, with multilabel stratified sampling in the ratio of 8:1:1, and retrained the model. In the training procedure, we applied the Adam optimizer [67] with the initial learning rate set to 2 × 10^−4^, and the exponential decay rate as default to 0.9 and 0.999, batch size 64. To make MIPPI pay more attention to those hard samples, categorical weighted focal loss [68] was used. Compared with classical cross-entropy loss, focal loss adds a modulating factor (γ = 2), which makes the function reduce the loss contribution from easy examples, resulting in increasing the importance of correcting misclassified samples:

$$\mathrm{FL}\left(p_{t}\right)=-a_{t}{\left(1-p_{t}y_{t}\right)}^{y}\text{log}\left(p_{t}\right)$$ (3)

where *p_t_* denotes prediction probability, *y_t_* denotes ground truth label, γ denotes modulating factor, and *a_t_* denotes a balanced variant.

Furthermore, different class weights ([0.25, 0.25, 0.1, 0.25]) were set to further ease the class imbalance problem. As mentioned above, only training data used hypothetical reverse mutation and no-mutation measure as a special data augmentation in our method. MIPPI model was implemented, trained, and tested using TensorFlow2.2 [69]. All the experiments were performed on a Linux system with NVIDIA Tesla V100 GPU. The tuning progress of MIPPI was performed through manual adjustments based on experience. The number of trainable parameters of MIPPI is 1,318,936.

As for the model evaluation metric, we chose 5 typical functions, including accuracy, precision, recall, F1 score, and MCC [70]:

$$\text{accuracy}=\frac{\mathrm{TP}+\mathrm{TN}}{\mathrm{TP}+\mathrm{TN}+\mathrm{FP}+\mathrm{FN}}$$ (4)

$$\text{precision}=\frac{\mathrm{TP}}{\mathrm{TP}+\mathrm{FP}}$$ (5)

$$\text{recall}=\frac{\mathrm{TP}}{\mathrm{TP}+\mathrm{FN}}$$ (6)

$$F_{1}=\frac{\mathrm{TP}}{\mathrm{TP}+\frac{1}{2}\left(\mathrm{FP}+\mathrm{FN}\right)}$$ (7)

$$\mathrm{MCC}=\frac{\mathrm{TP}\times \mathrm{TN}-\mathrm{FP}\times \mathrm{FN}}{\sqrt{\left(\mathrm{TP}+\mathrm{FP}\right)\left(\mathrm{TP}+\mathrm{FN}\right)\left(\mathrm{TN}+\mathrm{FP}\right)\left(\mathrm{TN}+\mathrm{FN}\right)}}$$ (8)

Here, *TP/TN/FP/FN* is the number of true positive/true negative/false positive/false negative for each class. F1 score and MCC are comprehensive metric functions for a multiclass classification task.

In a class imbalanced dataset, accuracy can be misleading as it only provides the general proportion of correct predictions but ignores the performance in each class. The other 4 metrics made up for this shortcoming, giving performance scores in each class to make a more detailed evaluation.

### Data augmentation

First, we generated a “reversed entry” by swapping the labels between the mutant and its unmutated protein from “decreasing,” “no effect,” and “increasing” entries in the initial training set so that the mutant protein would be labeled as an unmutated protein and the reference (original unmutated) would be labeled as a mutant to form a new pseudo entry. We then labeled these new pseudo entries as “increasing,” “no effect,” or “decreasing” correspondingly and added 10,999 new data entries to the initial training set (see Materials and Methods and Table S7). Next, we generated a “nonmutated entry” by using the unmutated sequence for the reference protein as well as the mutant sequence, pretending that mutations did not occur for all available entries in the initial MIPPI training data. We labeled these new pseudo entries as “no effect” and generated 16,451 new entries (Table S8). As a result, we managed to increase the MIPPI training set from an initial version of 16,451 entries to a modified version training set of 43,901 entries by using our modified data augmentation strategy and making the dataset more balanced (Table S8).

Considering the biological significance of the “disrupting” category, generating a hypothetical reverse category of “causing” or “increasing” from it might not be appropriate and biologically meaningful. Therefore, we excluded the “disrupting” category from the hypothetical reverse mutation strategy. To ensure data authenticity and prediction validity, we only applied hypothetical reverse mutation in the training set, not in the test set. Another easy-to-ignore issue is that proteins without mutations would cause “no effect” on the original PPI. Most studies did not consider this condition and excluded no-mutation sequences as input in similar tasks, such as ΔΔ*G* prediction. Although the reference protein will not cause PPI changes, various machine learning models have not considered this type and might output unexpected results if no corresponding sample data were included during training. In MIPPI, we added no-mutation samples with “no effect” labels for every original entry in the training set to learn a more robust distribution [27]. We generated “nonmutated entries” by using the unmutated sequence for the reference protein as well as the mutant sequence, pretending that mutations did not occur for all available entries in the initial MIPPI training data and labeling these new pseudo entries as “no effect.” Details of the data distribution after the data augmentation strategy are provided in Table S8.

### Multiple machine learning method comparison

LR, KNN [71], NB, RF [72], and XGBoost [73] were chosen in our comparison. Hyperparameters of these methods were optimized using the grid search function in PyCaret [74], with a 5-fold cross-validation performance to select the best hyperparameter set (Table S1). The data augmentation strategy was used in the training process of MIPPI in the comparison. Since we did not enhance the forward (original) mutation prediction capability of MIPPI through the data augmentation process (focused on reverse mutations and nonmutated entries), and MIPPI did not achieve any performance improvement due to data augmentation during the forward (original) mutation testing process (Table S10), the comparison was conducted under fair conditions.

### Details of ablation and input substitution test

First, we retrained the MIPPI by replacing the He uniform initialization layer with the Glorot uniform initialization layer, a commonly used default weight initialization method, at the model’s input and tested the model performance. For testing the GAP layer, we replaced it with a fully connected layer at the end of the model and retrained MIPPI. Next, we removed the PSSM profiles from the input of MIPPI and retrained the model to test its contribution to the prediction.

The partner protein branch was removed from the original MIPPI model structure to test the contribution of partner protein in model prediction. MIPPI-no partner was trained using the same protocol as MIPPI. The other 3 MIPPI variant models were implemented by modifying the partner branch input in the test set prediction. MIPPI-random partner input random amino acids to form an artificial partner protein sequence and input random PSSM data conforming to Gaussian distribution with mean and SD of all dataset PSSM matrices. MIPPI-random real partner input random paired partner protein sequence and PSSM in the training set. All the experiments were conducted on 5-fold cross-validation.

When evaluating the effectiveness of the transformer blocks, while preserving the other components, such as input branches and residual modules, we replaced the 3 transformer layers with 3 fully connected layers, 3 CNN layers, or one LSTM layers, respectively. The architectures and parameters of the alternative models used for comparison were empirically fine-tuned to maximize performance, and their performance was evaluated through a 5-fold cross-validation.

### Co-IP details

The SETD2-HA expression plasmid was received from the Lili laboratory. All mutated plasmids (S1624C, Y1666C, L1815W) were introduced using the Q5 Site-Directed Mutagenesis Kit (New England BioLabs), and mutations were confirmed via Sanger sequencing. The following primers were used:

SETD2-S1624C F: GGAAATTGCTgTCGTTTCATG

R: TTTTTGAGTGGCATCTATTATC

SETD2-Y1666C F: ACGTTTGACTgTCAGTTCCAG

R: TAACTCTGAGCCTGAAGG

SETD2-L1815W F: AAAAATATGTgGGAGGAAAGCAAAG

R: AGTAGGAATGGGCAAGTG

HEK293T cells were cultured in Dulbecco’s modified Eagle’s medium (DMEM), supplemented with 10% fetal bovine serum (FBS) and 1% penicillin/streptomycin at 37°C and 5% CO_2_. For transient transfection experiments, HEK293T cells were seeded in 6-well plates. The following day, cells were respectively transfected with plasmid WT, S1624C, Y1666C, and L1815W using Lipo2000 reagent (Invitrogen, Carlsbad, CA, USA). Then, 48 h after transfection, cells were subjected to the corresponding analysis.

HEK293T cells were lysed in 1× lysis buffer on ice for 30 min and centrifuged at 12,000 rpm for 15 min. The whole-cell lysates were incubated with primary antibody anti-HA at 4°C overnight. Then, the immunoprecipitated multiprotein complexes were pulled down with protein G beads (Beyotime, Shanghai, China) at 4°C for 2 h. The immunoprecipitated products were separated by SDS-PAGE and then detected by immunoblotting with indicated antibodies. Antibodies used in the study were anti-HA (Sigma, H9658), anti-SMAD3 (Absin, abs124584), and anti-TP53 (Absin, abs116840).

### SKEMPI v2 dataset preprocessing

SKEMPI v2 [75] is an independent dataset for model performance tests. Protein Data Bank (PDB) [76] identifiers in the dataset were mapped to PDB sequences, and all the features were calculated as mentioned above. As our model focused on binary PPI, entries with more than 2 chains were deleted. Multipoint mutant entries were dropped as well. Duplicated entries were all kept in the study because binding affinity from different references was quite different and no gold standard value was given. SKEMPI v2 provided temperature, affinities (*K*_d_) of the wild-type, and affinities of the mutant complexes. PPI binding affinity changes (ΔΔ*G*) upon mutation were calculated as:

$$\Delta \Delta G=\Delta G_{\mathrm{mut}}-\Delta G_{\mathrm{wt}}=\text{RTln}\left(\frac{Kd_{\mathrm{mut}}}{Kd_{\mathrm{wt}}}\right),$$ (9)

where *R* = 1.987 × 10^−3^kcal · K^−1^mol^−1^ is the gas constant, *T* is the temperature (K), and *K*d_mut_ and *K*d_wt_ are the equilibrium dissociation constant of mutant and wild-type protein–protein complexes, respectively.

### De novo mutation dataset preprocessing

The de novo mutations of DD and the control were downloaded from PsyMuKB (http://psymukb.net/). We chose the missense mutations with ANNOVAR annotation. As the input branch of the mutated protein of MIPPI only takes 50 amino acids close to mutation, we did not consider every transcript at the variant location, but the first transcript in the annotation. Corresponding protein sequences were retrieved by the biomaRt R package using RefSeq mRNA ID. PPIs for every mutation were acquired from the BioGRID database. Only physical PPIs with ≥2 reported pieces of evidence were chosen to guarantee PPI credibility. Protein sequences of BioGRID partner protein were acquired by a UniProt Retrieve/ID mapping search (https://www.uniprot.org/uploadlists/) using SwissProt ID. The Gene Ontology (GO) biological process enrichment of the DD-correlated genes was implemented using Metascape [77].

### Statistical validation of the relationship between attention weights and PPI interface

We downloaded the “Highest Confidence Interfaces” files from Interactome INSIDER [78] (http://interactomeinsider.yulab.org/) as the interface benchmark and mapped the output weights of the last self-attention layer of partner proteins to these PPI interfaces. The output weights of padding positions were discarded, and only the top 5 highest attention weights of each partner protein were selected. For each attention head of the self-attention layer, we performed a Mann–Whitney *U* test [79] to evaluate the statistical significance between the weights of interface residues and noninterface residues.

For the case study, we first extracted the crystal structure of Rac-p67^phox^ (https://www.rcsb.org/structure/1e96), a complex formed by binding between protein RAC1 and protein NCF2 and as part of a multiprotein enzyme complex that produces superoxide ions in response to microbial infection, from PDB. We then visualized the structure of Rac-p67^phox^ using PYMOL [80], with residues in different colors indicating different attention weights mapped. These attention weights were divided into 3 categories based on their magnitude, with values more than 80 being considered high weights, between 25 and 80 as median weights, and less than 25 as low weights.

### Software implementation

The tools and packages used in this study include Python version 3.7, NumPy version 1.19, pandas version 1.0, TensorFlow version 2.2, Matplotlib version 3.1, seaborn version 0.11, and SciPy version 1.3. MIPPI is hosted on our web server https://www.psymukb.net/MIPPI.

## Data Availability

The source code of MIPPI is available at https://github.com/kentergav/MIPPI.
