# Supplementary material for: Inferring the Effects of Protein Variants on Protein–Protein Interactions with Interpretable Transformer Representations
Source: Research (Wash D C). 2023 Sep 11;6:0219. doi: 10.34133/research.0219 (PMC10494974; doi:10.34133/research.0219)
Supplement: Supplementary 1 — Figs. S1 to S9 Tables S1 to S12 [file research.0219.f1.docx]

# Supplementary Materials

Inferring the Effects of Protein Variants on Protein–protein Interactions with Interpretable Transformer Representations

Supplementary Figure 1: Visualization of the representation ability of MIPPI.

Supplementary Figure 2: Attention weights visualization of mutated proteins.

Supplementary Figure 3: Model evaluation on SKEMPI v2.

Supplementary Figure 4: Data preprocessing.

Supplementary Figure 5: Original transformer encoder structure (left) and modified transformer encoder structure in MIPPI.

Supplementary Figure 6: The reversed entries used in the data augmentation strategy.

Supplementary Figure 7: The GO biological processes enrichment results of DD-correlated mutated proteins from the PsyMuKB dataset.

Supplementary Figure 8: Case study of PDB ID:1E96.

Supplementary Table 1: Optimized hyperparameters of multiple machine-learning methods.

Supplementary Table 2: Machine learning methods estimation in binary classification.

Supplementary Table 3: Model performance of the ablation study.

Supplementary Table 4: Block number 5-fold cross-validation performance.

Supplementary Table 5: Degree of contradictory label level.

Supplementary Table 6: IMEx dataset preprocessing details.

Supplementary Table 7: Hypothetical reverse mutation transition rule.

Supplementary Table 8: Processed data set category distribution statistics.

Supplementary Table 9: SKEMPI v2 dataset class distribution by MIPPI prediction.

Supplementary Table 10: Performance of MIPPI trained with/without data augmentation.

Supplementary Table 11. The number of mutations in the categories predicted by MIPPI prediction.

Supplementary Table 12. The detailed GO biological processes enrichment results of DD-correlated mutated proteins from the PsyMuKB dataset.

### Supplementary Figure 1


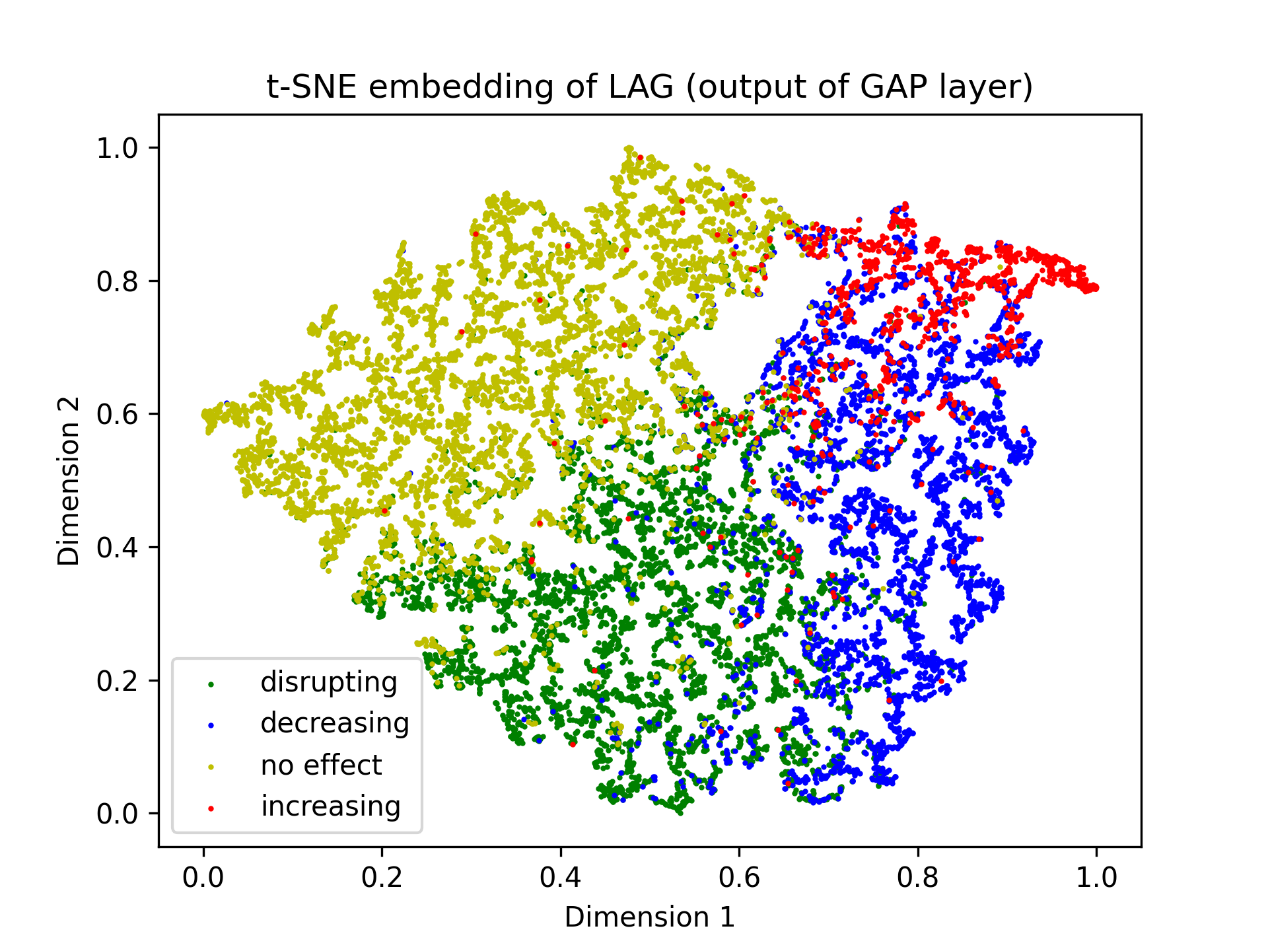


Supplementary Figure 1. t-SNE embedding of the model output of the GAP layer (LAG: layer output after GAP) represents good representation power of MIPPI in a four-class prediction task. The red points represent the “increasing” entries, the blue points represent the “decreasing” entries, the green points represent the “disrupting” entries, and the yellow points represent the “no effect” entries.

### Supplementary Figure 2


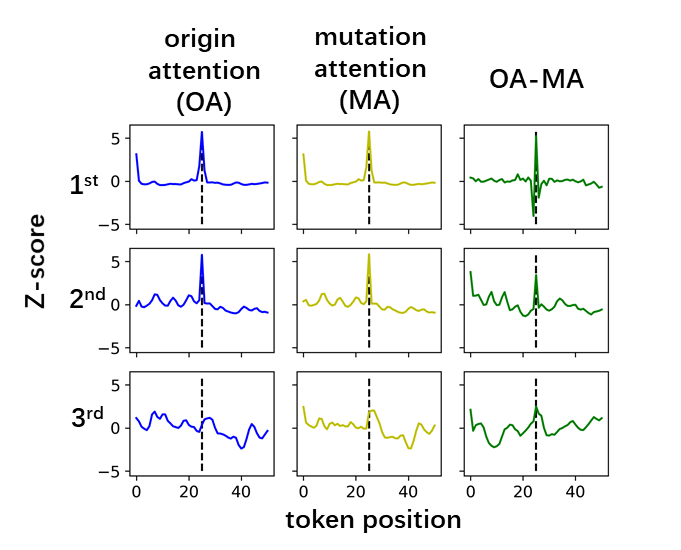


Supplementary Figure 2. Average attention weights visualization of the mutant part. Attention weights focus on the mutant position, and as the stage progresses, other positions get higher weights gradually.

### Supplementary Figure 3


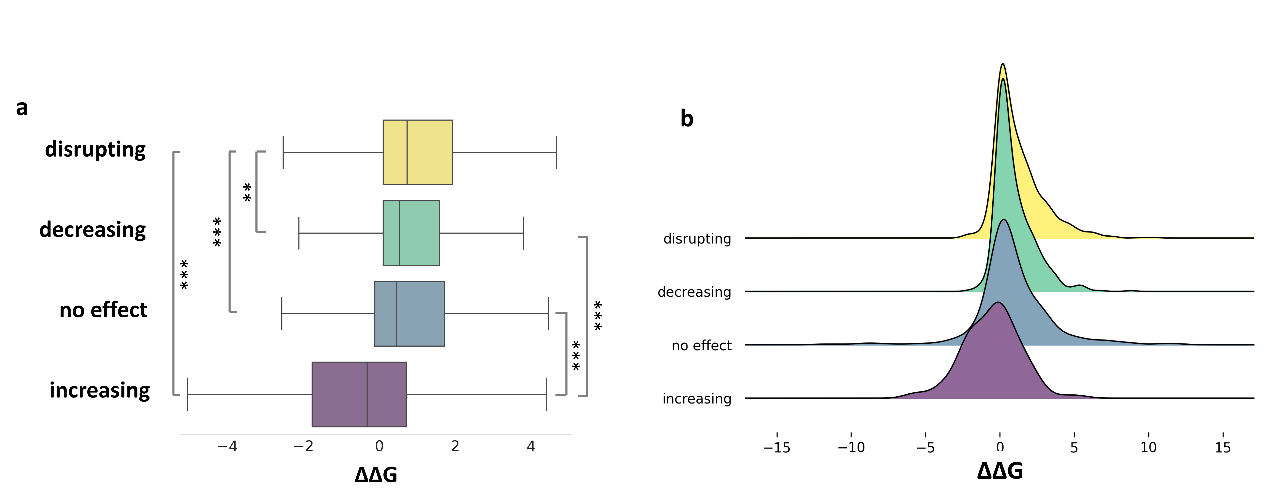


Supplementary Figure 3. a The mapping between prediction results of four impact outcomes categories (Y-axis) and ΔΔG values (X-axis) annotated in SKEMPI v2, when given the same input data for analysis. The values of ΔΔG binding affinities were shown from high to low as negative to positive (x-axis). ANOVA tests were performed between each pair of categories for the significance test. Stars indicate that all presented p-values of ANOVA tests were Bonferroni corrected (**:<0.01, ***: <0.001). As expected, we observed ΔΔG values, ranging from negative to positive, were highly correlated with MIPPI’s classification results: the “disrupting” class showed the highest ΔΔG mean values (1.21), closely followed by the “decreasing” class (0.96) and the “no effect” class (0.84), and the“increasing” class achieved the lowest mean ΔΔG value (-0.52 kcal/mol). b ΔΔG probability distribution with MIPPI prediction results on SKEMPI v2. Consistent with ΔΔG mean value, in the probability distribution, and “increasing” is significantly inclined left. Other classes concentrate on positive values near zero, and the distribution gradually moves to the right as the class impact worsens. We noticed that the mean value of ΔΔG from the SKEMPI v2 dataset was 0.98 $\text{kcal}\text{/mol}$, indicating that more entries in SKEMPI decreased the interaction strength of the original PPIs than other impact types. The observation was consistent with the prediction results of MIPPI using SKEMPI data, with more negative effects (“decreasing” and “disrupting”) predicted on the original PPIs (Supplementary Table 9) than positive effects.

### Supplementary Figure 4


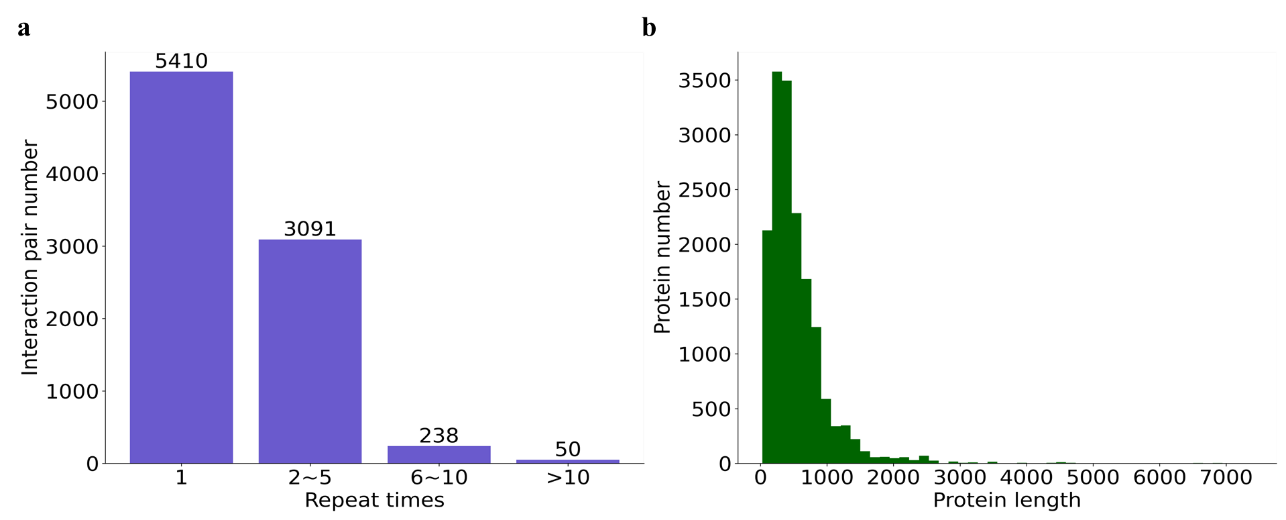


Supplementary Figure 4. Data preprocessing. **a** count the protein pair duplicated times for redundancy check, only unique items were chosen. **b** visualization of partner protein length distribution, over 90% of partner proteins are shorter than 1024 AA.

### Supplementary Figure 5


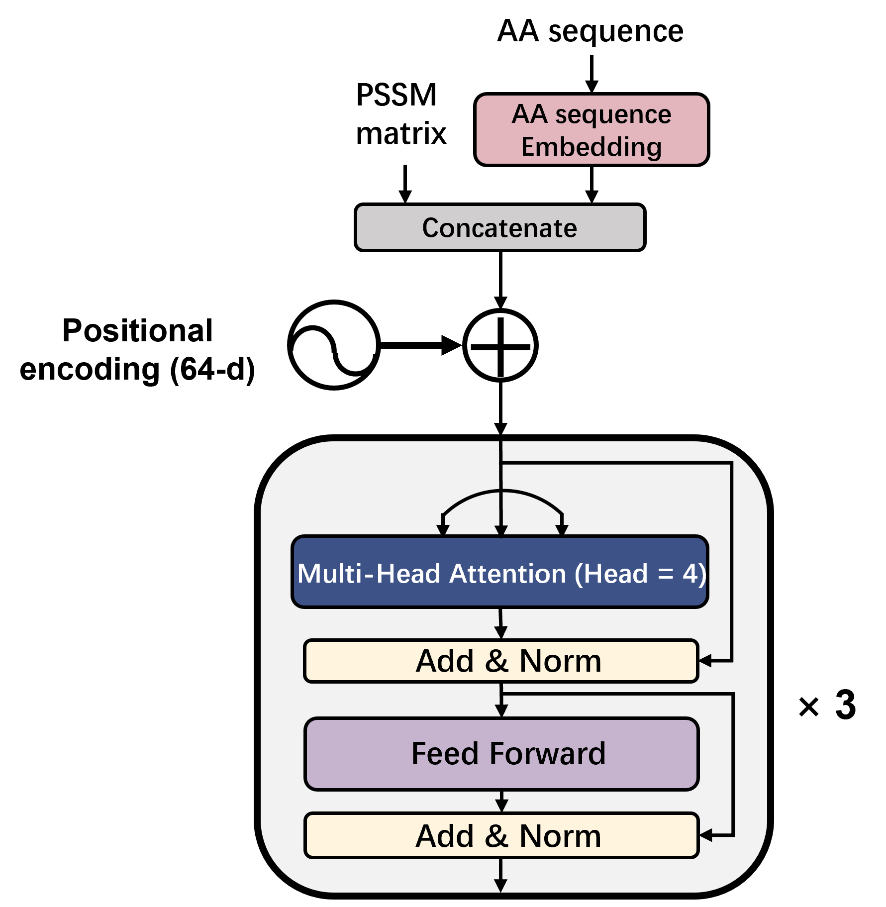


Supplementary Figure 5. Original transformer encoder structure (left) and modified transformer encoder structure in MIPPI (right). MIPPI concatenates AA sequence embedding and PSSM matrix to form the entire input embedding representation.

### Supplementary Figure 6


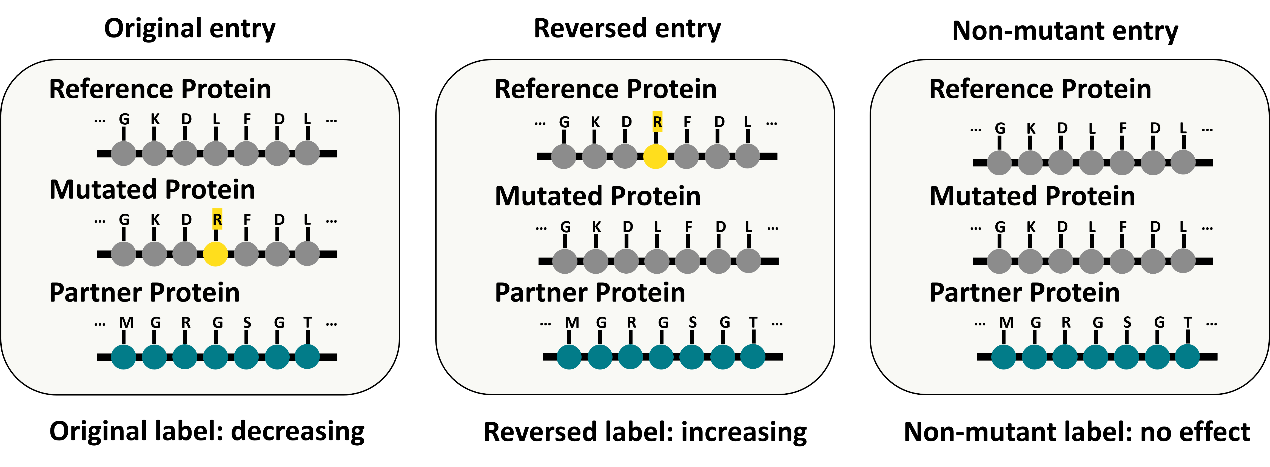


Supplementary Figure 6. The“reversed entry” was generated by swapping the labels between the mutant and its unmutated protein from “decreasing”, “no effect” and “increasing” entries in the initial training set, so that the mutant protein would be labeled as an unmutated protein and the reference (original unmutated) would be labeled as a mutant to form a new pseudo entry. We then labeled these new pseudo entries as “increasing”, “no effect” or “decreasing” correspondingly (see Methods and Supplementary Table 7). The“non-mutated entry” was generated by using the unmutated sequence for both the reference protein and the mutant sequence, pretending that mutations did not occur for all available entries. We labeled these new pseudo entries as “no effect”.

### Supplementary Figure 7


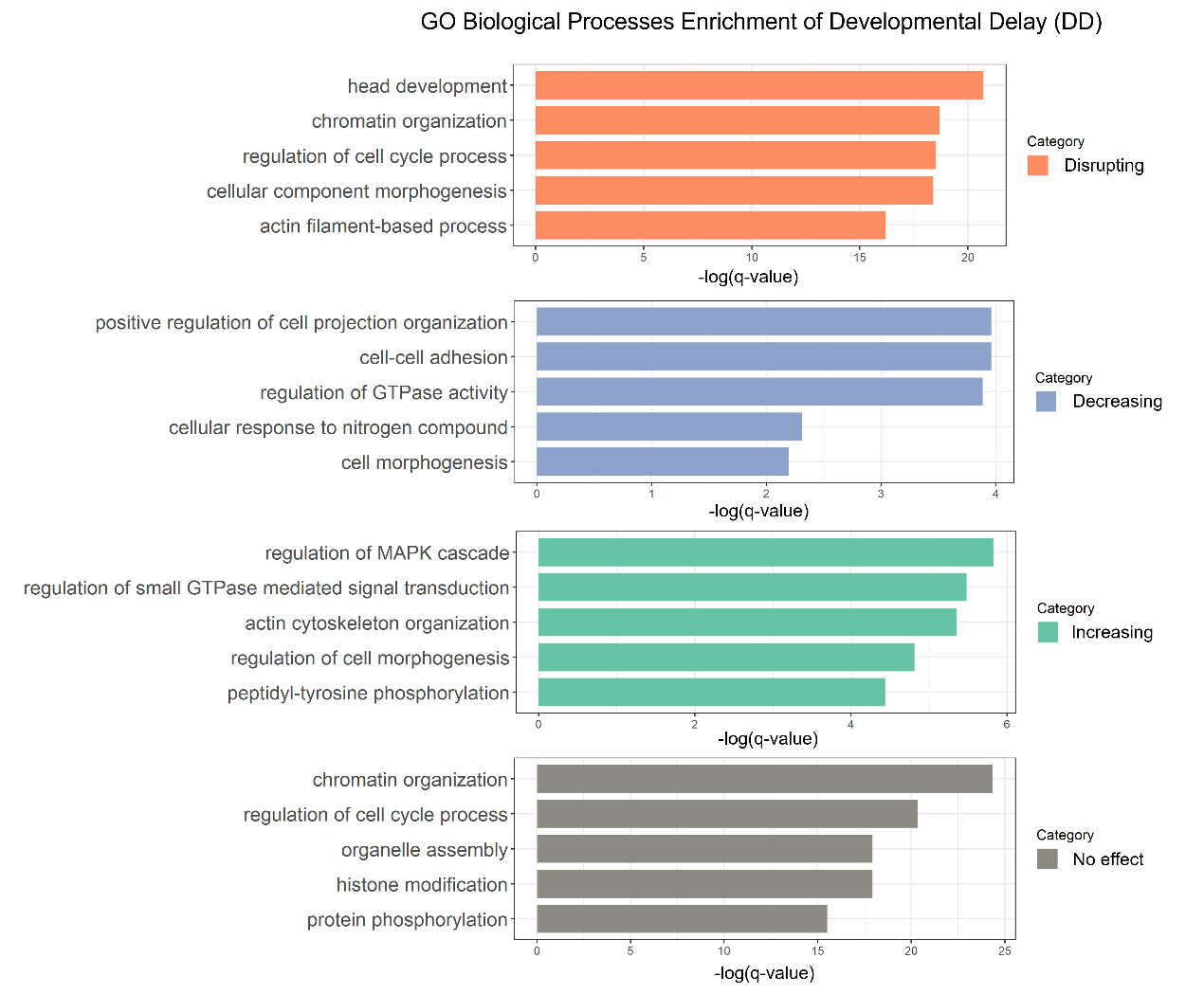


Supplementary Figure 7: The GO biological processes enrichment results of DD-correlated mutated proteins from the PsyMuKB dataset. The categories were predicted by MIPPI, and the enrichment results were implemented using Metascape.

### Supplementary Figure 8


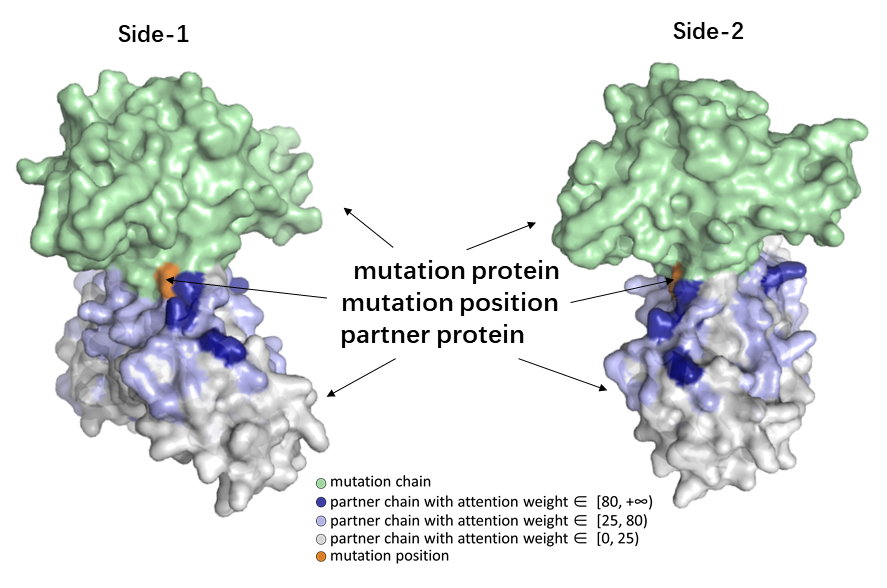


Supplementary Figure 8. A human signaling protein as an illustration of attention weights distribution. Protein Data Bank ID for the structure shown is 1E96. The mutation chain is 1E96_A (UniProtKB accession number: P63000), and the partner chain is 1E96_B (UniProtKB accession number: P19878). The model weights were generated from the last self-attention layer of MIPPI with the second attention head. The 3D structure of the protein and its coloring were derived from PYMOL.

### Supplementary Figure 9


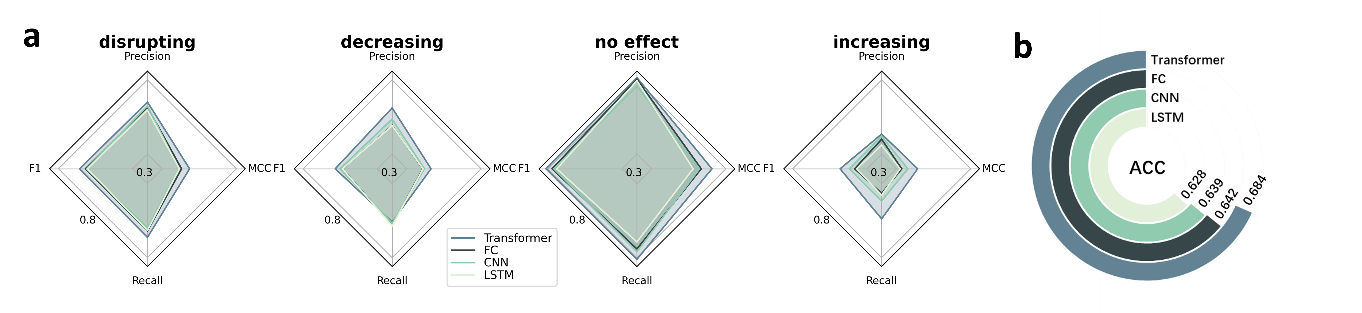


Supplementary Figure 9. The ablation study of the MIPPI structure. The Transformer blocks of MIPPI was replaced by fully-connected layers, CNN layers and LSTM layers. The Transformer blocks used in MIPPI outperformed all other compared components.

### Supplementary Table 1

Supplementary Table 1. Optimized hyperparameters of multiple machine-learning methods.

| Logistic regression | linear_model.LogisticRegression(C=1.021, class_weight=None, dual=False, fit_intercept=True, intercept_scaling=1, l1_ratio=None, max_iter=100, multi_class='auto', n_jobs=None, penalty='l2', random_state=139, solver='lbfgs', tol=0.0001, verbose=0, warm_start=False) |
| --- | --- |
| kNN | KNeighborsClassifier(algorithm='auto', leaf_size=30, metric='manhattan', metric_params=None, n_jobs=-1, n_neighbors=12, p=2, weights='distance') |
| Naïve Bayes | naive_bayes.GaussianNB(priors=None, var_smoothing=0.003) |
| Random forest | RandomForestClassifier(bootstrap=False, ccp_alpha=0.0, class_weight=None, criterion='gini', max_depth=50, max_features='sqrt', max_leaf_nodes=None, max_samples=None, min_impurity_decrease=0.0, min_impurity_split=None, min_samples_leaf=4, min_samples_split=7, min_weight_fraction_leaf=0.0, n_estimators=70, n_jobs=-1, oob_score=False, random_state=0, verbose=0, warm_start=False) |
| XGBoost | XGBClassifier(base_score=0.5, booster='gbtree', colsample_bylevel=1, colsample_bynode=1, colsample_bytree=1, gamma=0, gpu_id=-1, importance_type='gain', interaction_constraints='', learning_rate=0.1, max_delta_step=0, max_depth=10, min_child_weight=1, monotone_constraints='()', n_estimators=200, n_jobs=-1, num_class=4, num_parallel_tree=1, objective='multi:softprob', random_state=139, reg_alpha=0, reg_lambda=1, scale_pos_weight=None, subsample=0.9, tree_method='exact', validate_parameters=1, verbosity=0) |

### Supplementary Table 2

Supplementary Table 2. MIPPI and multiple machine learning methods for binary classification estimation on a 5-fold cross-validation.

|  | **class** | **precision** | **recall** | **F1-score** | **MCC** | **ACC** |
| --- | --- | --- | --- | --- | --- | --- |
| **logistic regression** | negative | 47.5 | 94.0 | 63.1 | 36.9 | 59.9 |
|  | positive | 92.2 | 40.4 | 56.1 |  |  |
| **kNN** | negative | 58.5 | 94.9 | 72.4 | 55.1 | 73.6 |
|  | positive | 95.4 | 61.3 | 74.7 |  |  |
| **naïve bayes** | negative | 42.7 | 78.8 | 55.4 | 18.5 | 53.6 |
|  | positive | 76.3 | 39.2 | 51.8 |  |  |
| **random forest** | negative | 56.0 | **96.1** | 70.8 | 52.5 | 71.1 |
|  | positive | **96.2** | 56.7 | 71.3 |  |  |
| **XGBoost** | negative | 67.6 | 91.1 | 77.6 | 63.6 | 80.8 |
|  | positive | 93.6 | 74.9 | 83.2 |  |  |
| **MIPPI-binary** | negative | **83.0** | 79.5 | **81.2** | **70.8** | **86.6** |
|  | positive | 88.5 | **90.7** | **89.6** |  |  |

### Supplementary Table 3

Supplementary Table 3. Model performance of the ablation study.

| **MIPPI type** | **class** | **precision** | **recall** | **F1-score** | **MCC** | **ACC** |
| --- | --- | --- | --- | --- | --- | --- |
| MIPPI | disrupting | **65.1** | **66.3** | **65.7** | **48.4** | **68.4** |
|  | decreasing | **61.1** | 55.9 | **58.4** | **46.2** |  |
|  | no effect | **81.6** | **81.1** | **81.3** | **70.6** |  |
|  | increasing | **43.5** | **53.8** | **48.0** | **44.3** |  |
| no PSSM | disrupting | 62.1 | 65.0 | 63.5 | 44.9 | 66.1 |
|  | decreasing | 59.9 | 52.8 | 56.1 | 43.7 |  |
|  | no effect | 80.3 | 78.9 | 79.5 | 68.0 |  |
|  | increasing | 37.6 | 48.4 | 42.2 | 38.0 |  |
| Glorot uniform | disrupting | 64.6 | 62.8 | 63.7 | 46.1 | 67.3 |
|  | decreasing | 59.1 | 56.8 | 57.9 | 45.1 |  |
|  | no effect | 80.0 | **81.1** | 80.5 | 69.3 |  |
|  | increasing | 42.8 | 51.0 | 46.4 | 42.5 |  |
| replace GAP to fully-connected layer | disrupting | 63.1 | 65.8 | 64.4 | 46.2 | 67.0 |
|  | decreasing | 60.7 | 53.7 | 56.9 | 44.7 |  |
|  | no effect | 80.6 | 79.9 | 80.2 | 69.0 |  |
|  | increasing | 40.2 | 49.7 | 44.4 | 40.4 |  |
| MIPPI-no partner  (model retrained) | disrupting | 58.6 | 60.5 | 59.5 | 39.0 | 62.2 |
|  | decreasing | 51.5 | 44.3 | 47.6 | 33.0 |  |
|  | no effect | 75.2 | 78.0 | 76.6 | 62.7 |  |
|  | increasing | 43.4 | 49.0 | 45.9 | 42.0 |  |
| MIPPI-random partner | disrupting | 43.0 | 65.2 | 51.6 | 20.9 | 45.5 |
|  | decreasing | 37.0 | 33.9 | 34.9 | 16.2 |  |
|  | no effect | 79.1 | 34.8 | 47.9 | 38.5 |  |
|  | increasing | 28.1 | 48.3 | 35.2 | 30.8 |  |
| MIPPI-random real partner | disrupting | 46.3 | 56.3 | 50.7 | 22.9 | 47.5 |
|  | decreasing | 34.4 | 29.5 | 31.7 | 12.5 |  |
|  | no effect | 63.9 | 52.7 | 57.7 | 37.4 |  |
|  | increasing | 28.6 | 39.7 | 33.1 | 28.0 |  |
| replace Transformer to fully-connected layers | disrupting | 61.7 | 62.0 | 61.9 | 43.0 | 64.2 |
|  | decreasing | 49.3 | 58.3 | 53.4 | 40.6 |  |
|  | no effect | 80.5 | 74.1 | 77.2 | 63.4 |  |
|  | increasing | 40.0 | 36.5 | 38.2 | 33.6 |  |
| replace Transformer to CNN | disrupting | 62.4 | 59.9 | 61.1 | 41.3 | 63.9 |
|  | decreasing | 53.6 | 56.8 | 55.2 | 41.7 |  |
|  | no effect | 75.9 | 76.0 | 76.0 | 62.2 |  |
|  | increasing | 42.0 | 41.7 | 41.8 | 37.7 |  |
| replace Transformer to LSTM | disrupting | 59.5 | 61.6 | 60.5 | 41.5 | 62.8 |
|  | decreasing | 49.1 | **58.6** | 53.5 | 40.8 |  |
|  | no effect | 79.5 | 69.4 | 74.1 | 57.9 |  |
|  | increasing | 35.9 | 37.7 | 36.8 | 32.5 |  |

Numbers in bold indicate the best experimental results.

### Supplementary Table 4

Supplementary Table 4. Block number vs. 5-fold cross-validation performance.

| **encoder block num** | **class** | **precision** | **recall** | **F1-score** | **MCC** | **ACC** | **top2 ACC** |
| --- | --- | --- | --- | --- | --- | --- | --- |
| 1-block | disrupting | 63.1 | 65.6 | 64.3 | 46.1 | 67.1 | 85.6 |
|  | decreasing | 59.7 | 53.3 | 56.2 | 43.7 |  |  |
|  | no effect | 79.3 | 81.8 | 80.4 | 69.0 |  |  |
|  | increasing | 43.5 | 42.7 | 42.9 | 39.1 |  |  |
| 2-block | disrupting | 65.3 | 65.2 | 65.2 | 48.0 | 67.8 | 87.4 |
|  | decreasing | 59.8 | 54.4 | 56.9 | 44.4 |  |  |
|  | no effect | 80.2 | 82.4 | 81.3 | 70.3 |  |  |
|  | increasing | 41.6 | 48.6 | 44.7 | 40.7 |  |  |
| 3-block | disrupting | **65.1** | **66.3** | **65.7** | **48.4** | **68.4** | 88.5 |
|  | decreasing | **61.1** | 55.9 | 58.4 | **46.2** |  |  |
|  | no effect | **81.6** | **81.1** | **81.3** | **70.6** |  |  |
|  | increasing | 43.5 | 53.8 | 48.0 | 44.3 |  |  |
| 4-block | disrupting | 64.3 | 64.0 | 64.1 | 46.4 | 67.6 | **88.6** |
|  | decreasing | 60.1 | **57.2** | **58.6** | 46.1 |  |  |
|  | no effect | 80.3 | 80.0 | 80.1 | 68.8 |  |  |
|  | increasing | **45.2** | 54.3 | **49.3** | **45.6** |  |  |

### Supplementary Table 5

Supplementary Table 5. Degree of contradictory label level.

| **Level** | **Label 1** | **Label 2** |
| --- | --- | --- |
| Mild | Disrupting | Decreasing |
|  | Decreasing | No effect |
|  | No effect | Increasing |
| Middle | Disrupting | No effect |
|  | Decreasing | Increasing |
| Bad | Disrupting | Increasing |

*Label 1 and label 2 could be exchanged.

### Supplementary Table 6

Supplementary Table 6. IMEx dataset preprocessing details.

| **STEPS** | **remaining items** |
| --- | --- |
| 1. Raw | 58,250 |
| 1. Drop high-throughput data | 47,222 |
| 1. Select binary protein-protein interaction | 43,126 |
| 1. Drop items with different number of partners and proteinAC | 35,267 |
| 1. Drop PPI with multi-mutated proteins | 34,712 |
| 1. Drop proteins without Uniprot AC | 34,696 |
| 1. Drop items with "mutation" type | 31,791 |
| 1. Drop "PRO_" protein items | 31,488 |
| 1. Merge multi-point items into one | 26,861 |
| 1. Drop items with the same participants (for unknown mutated or wildtype in the interaction) | 23,860 |
| 1. Drop items with rare amino acid | 23,819 |
| 1. Drop multi-point items | 21,390 |
| 1. Drop duplicated items (keep once) | 17,713 |
| 1. Drop conflict items (keep none) | 16,573 |
| 1. Drop items without valid PSSM | 16,505 |
| 1. Drop "Causing" items | 16,451 |

### Supplementary Table 7

Supplementary Table 7. Hypothetical reverse mutation transition rule.

| Original category | hypothetical reverse category |
| --- | --- |
| Disrupting | - |
| Decreasing | Increasing |
| No effect | No effect |
| Increasing | Decreasing |

### Supplementary Table 8

Supplementary Table 8. Processed data set category distribution statistics.

|  | Disrupting | Decreasing | No effect | Increasing |
| --- | --- | --- | --- | --- |
| Original data | 5452 | 3920 | 6000 | 1079 |
| Hypothetical reverse data | - | 1079 | 6000 | 3920 |
| no-mutation data | - | - | 16451 | - |
| Total | 5452 | 4999 | 28451 | 4999 |

### Supplementary Table 9

Supplementary Table 9. SKEMPI v2 dataset class distribution by MIPPI prediction.

|  | **Disrupting** | **Decreasing** | **No effect** | **Increasing** |
| --- | --- | --- | --- | --- |
| **Original dataset** | 1416 | 712 | 619 | 203 |
| **Hypothetical reverse mutations** | 338 | 110 | 819 | 1683 |

### Supplementary Table 10

Supplementary Table 10. Performance of MIPPI trained with/without data augmentation.

| **test set** | **training strategy** | **class** | **precision** | **recall** | **F1-score** | **MCC** | **ACC** |
| --- | --- | --- | --- | --- | --- | --- | --- |
| original | DA | disrupting | 65.1 | 66.3 | 65.7 | 48.4 | 68.4 |
|  |  | decreasing | 61.1 | 55.9 | 58.4 | 46.2 |  |
|  |  | no effect | 81.6 | 81.1 | 81.3 | 70.6 |  |
|  |  | increasing | 43.5 | 53.8 | 48.0 | 44.3 |  |
| original | no DA | disrupting | 61.6 | 68.3 | 64.7 | 46.0 | 68.4 |
|  |  | decreasing | 57.9 | 62.8 | 60.2 | 47.2 |  |
|  |  | no effect | 85.0 | 75.1 | 79.7 | 69.5 |  |
|  |  | increasing | 66.5 | 51.0 | 57.6 | 55.7 |  |
| reverse | DA | disrupting | - | - | - | - | 77.1 |
|  |  | decreasing | 51.0 | 50.2 | 50.5 | 45.2 |  |
|  |  | no effect | 90.1 | 86.4 | 88.2 | 74.7 |  |
|  |  | increasing | 81.4 | 70.2 | 75.4 | 63.7 |  |
| reverse | no DA | disrupting | - | - | - | - | 44.2 |
|  |  | decreasing | 9.7 | 26.8 | 14.2 | 0.00 |  |
|  |  | no effect | 93.0 | 73.6 | 82.2 | 67.3 |  |
|  |  | increasing | 20.4 | 3.9 | 6.6 | -9.62 |  |
| no mutation | DA | no effect | 100.0 | 100.0 | 100.0 | - | 100.0 |
| no mutation | no DA | no effect | 100.0 | 31.9 | 48.3 | - | 31.9 |

The “disrupting” entries are excluded from hypothetical reverse mutation, so metrics in “disrupting” of the reverse test set is “-”. The “no mutation” set only contains entries in the “no effect” label, $TN=FP=0$ and the denominator of MCC became infinite, shown in “-”.

### Supplementary Table 11

Supplementary Table 11. The number of mutations in the categories predicted by MIPPI prediction.

|  | **Disrupting** | **Decreasing** | **No effect** | **Increasing** |
| --- | --- | --- | --- | --- |
| **developmental delay (DD)** | 1,148 | 193 | 1,647 | 200 |
| **Control** | 315 | 60 | 605 | 60 |

### Supplementary Table 12

Supplementary Table 12. The detailed GO biological processes enrichment results of DD-correlated mutated proteins from the PsyMuKB dataset.

| **Category** | **Term** | **Description** | **logP** | **-log(q-value)** |
| --- | --- | --- | --- | --- |
| **decreasing** | GO:0031346  GO:0098609  GO:0043087 | positive regulation of cell projection organization  cell-cell adhesion  regulation of GTPase activity | -8.07  -8.02  -7.73 | -3.96  -3.96  -3.89 |
| **disrupting** | GO:0060322  GO:0006325  GO:0010564  GO:0032989  GO:0030029  GO:0006259  GO:0034330  GO:0016570  GO:0007507  GO:0000278  GO:0061061  GO:0120035 | head development  chromatin organization  regulation of cell cycle process  cellular component morphogenesis  actin filament-based process  DNA metabolic process  cell junction organization  histone modification  heart development  mitotic cell cycle  muscle structure development  regulation of plasma membrane bounded cell projection organization | -25.06  -22.28  -22.04  -21.86  -19.44  -18.04  -17.80  -17.15  -16.02  -15.61  -15.01  -14.96 | -20.73  -18.70  -18.53  -18.41  -16.21  -15.03  -14.81  -14.31  -13.26  -12.87  -12.33  -12.31 |
| **increasing** | GO:0043408  GO:0051056  GO:0030036  GO:0022604  GO:0018108  GO:0070925  GO:0045787 | regulation of MAPK cascade  regulation of small GTPase mediated signal transduction  actin cytoskeleton organization  regulation of cell morphogenesis  peptidyl-tyrosine phosphorylation  organelle assembly  positive regulation of cell cycle | -10.18  -9.14  -8.86  -8.05  -7.53  -6.96  -6.36 | -5.82  -5.48  -5.36  -4.81  -4.44  -4.00  -3.55 |
| **no effect** | GO:0006325  GO:0010564  GO:0070925  GO:0016570  GO:0006468  GO:0060322  GO:0043009  GO:0032446  GO:0061061  GO:0000278  GO:0032989  GO:0030029 | chromatin organization  regulation of cell cycle process  organelle assembly  histone modification  protein phosphorylation  head development  chordate embryonic development  protein modification by small protein conjugation  muscle structure development  mitotic cell cycle  cellular component morphogenesis  actin filament-based process | -28.71  -24.24  -21.54  -21.50  -18.90  -18.69  -18.68  -18.37  -18.09  -17.65  -17.34  -15.17 | -24.35  -20.36  -17.93  -17.93  -15.51  -15.44  -15.44  -15.19  -14.97  -14.60  -14.34  -12.33 |
